# Awareness of Oral Cancer Among Users of Smokeless Tobacco: A Cross-Sectional Study

**DOI:** 10.7759/cureus.50404

**Published:** 2023-12-12

**Authors:** Fahd Alharbi, Hatim Alsaedi, Nader S Alharbi, Rawan Alharbi, Hussain Alharbi, Abdullah Alazmi, Fahad Alghamdi

**Affiliations:** 1 Department of Otolaryngology, Head and Neck Surgery, Taibah University, Madinah, SAU; 2 Medical School, Shaqra University, Shaqra, SAU; 3 Medical School, King Abdulaziz University Faculty of Medicine, Rabigh, Jeddah, SAU; 4 Medical School, College of Medicine, Qassim University, Buraydah, SAU; 5 Department of Otolaryngology, Head and Neck Surgery, Al-Baha University, Al-Baha, SAU

**Keywords:** tobacco, cancer, oral mucosal ulcers, oral cancer, smokeless tobacco

## Abstract

Introduction: Smokeless tobacco (SLT) stands out for its higher nicotine absorption and its role in preventable fatalities. The Global Adult Tobacco survey in Saudi Arabia revealed SLT usage, while past legislation restricted its use. Linking SLT consumption to oral cancer and oral mucosal ulcers, the study addresses its prevalence in head and neck malignancies.

Methodology: This study is cross-sectional and includes adult users of SLT. Raosoft (Raosoft Inc., Seattle, WA) was used to calculate the sample size. The data was analyzed using SPSS software (IBM Corp., Armonk, NY).

Results: The research study investigated various sociodemographic characteristics and prevalence of SLT use among participants. All participants reported using SLT, with toombak (33.2%) and shamma (36.0%) being the most prevalent. Notably, reasons for initiating SLT included influence from peer pressure (33.6%), alternatives to smoking (32.0%), and influence from relatives (19.0%). While 75.1% intended to quit within a year, awareness of SLT’s harmfulness varied: 40.3% believed it was less harmful than smoking, and 57.7% recognized its link to oral cancer. Additionally, 62.2% believed SLT could lead to dependence. Sociodemographic factors generally did not significantly affect awareness of SLT causing oral cancer.

Conclusion: The findings indicate a significant prevalence of SLT use, with toombak and shamma being the most common types consumed. Awareness of the potential harm of SLT use in relation to oral cancer varied among participants, with a notable proportion misunderstanding its harmfulness compared to smoking tobacco.

## Introduction

Tobacco is an agricultural substance obtained from the leaves of the Nicotiana genus [1]. The tobacco plant contains an alkaloid component known as nicotine, and how much of it is absorbed by the body depends on several factors, including the type of tobacco used, whether it is smoked or consumed in a smokeless form, and whether a filter is present [2].

In comparison to smoke, smokeless tobacco (SLT) has a higher rate of nicotine absorption [1,3]. Tobacco product usage is one of the leading causes of preventable fatalities, accounting for more than 6 million deaths annually across the globe [4].

The Kingdom of Saudi Arabia conducted the Global Adult Tobacco Survey (GATS) in 2019, a nationally representative survey that followed a defined protocol. A total of 11,381 individual interviews were conducted, and 2.4% of them currently use SLT [5]. However, in 1990, legislation was introduced to restrict the use of SLT products in KSA [6].

Consuming SLT products could promote oral cancer and malignant lesions of the oral cavity [7-8]. Recent studies have found a significant correlation between SLT usage and the presence of oral mucosal ulcers [9-10]. All tobacco products, including SLT, are recognized as significant risk factors for oral cancer [11]. About 26% of all head and neck malignancies diagnosed annually in Saudi Arabia are malignant oral cavity tumors [12]. The majority of the consequences of oral cavity malignancies can be avoided by avoiding identified risk factors and also by detecting them early [13-14]. Delayed diagnosis of oral cancer leads to greater treatment morbidity and lower survival rates. According to Al-Maweri et al., the general Saudi population has a general lack of understanding of the indicators and risk factors for oral cancer, which may cause a delay in diagnosis and treatment. Numerous studies have established that smokers in Saudi Arabia lack knowledge of oral cancer. However, no further studies have been conducted on SLT users, even though they are at a higher risk of developing oral cancer [15]. Assessing the awareness and knowledge of oral cancer among SLT users in Saudi Arabia can help in conducting healthcare campaigns to educate those individuals regarding oral cancer, which in turn will aid in the prevention, early diagnosis, and treatment of the disease. The aim of this study is to evaluate oral cancer knowledge among users of SLT in Saudi Arabia.

## Materials and methods

In this study, we obtained ethical approval from Taibah University to conduct a cross-sectional examination of oral cancer knowledge among adults who use SLT products in Saudi Arabia. Our study focused on adult residents of Saudi Arabia who use SLT products, with those under 18 years old and those who use smoking tobacco being excluded. Data was collected over two months through a Google Forms questionnaire, which included 27 questions organized into three categories: participants' demographic and background information, the prevalence and consumption patterns of SLT, and awareness regarding oral cancer.

To ensure the questionnaire's relevance and validity, we drew from existing research [2, 6, 16-18]. We conducted data collection anonymously, and participation was entirely voluntary. Participants completed self-administered surveys, and we provided information about the study's objectives and the confidentiality of the questionnaire to ensure participants' understanding and compliance with ethical standards.

Data analysis involved both descriptive statistics, such as calculating frequencies and percentages, and inferential statistics, including Fisher's exact test. We applied a significance level of p ≤ 0.05 with a 95% confidence interval for determining statistical significance. IBM SPSS software v.27.0.1 (IBM Corp., Armonk, NY) was used for data analysis. Our dataset included 253 responses. Importantly, this project was unfunded, and there were no conflicts of interest among the participants. The research findings will be presented in a research report and may be shared at conferences and published in scientific journals. We also acknowledge potential limitations in the study, including the possibility of response bias and the use of Google Forms for data collection.

## Results

Table 1 presents the sociodemographic characteristics of the study participants. Regarding age distribution, the largest proportion of participants falls within the 18-24 year age group, comprising 32.4% of the sample, followed by the 25-34 year age group at 31.6%. Successively smaller proportions are distributed among the older age groups, with the 35-44 year age group accounting for 17.4%, 45-54 year age group accounting for 10.7%, 55-64 year age group accounting for 4.7%, and participants aged over 65 constituting 3.2% of the sample. Gender distribution reveals that the majority of participants identify as male, making up 77.1% of the sample, while the remaining 22.9% identify as female. Educational status indicates a diverse range of participants. The distribution shows that the highest number of respondents received education at the university level and above, making up 61.3% of the sample, while 26.5% completed high school, 6.7% finished middle school, and lower proportions of the population received primary schooling (2.0%) or no education (3.6%). Marital status highlights that a significant proportion of participants are currently single, representing 56.1% of the sample, followed by those who are currently married at 39.5%, and a smaller group of divorced or widowed individuals at 4.3%. Employment status reveals that the majority of participants are employed, accounting for 55.7% of the sample, while the remaining 44.3% are unemployed. Nationality distribution illustrates that the majority of participants are Saudi nationals (90.1%), with smaller numbers of individuals representing other nationalities, such as Egyptian (1.6%), Syrian (1.2%), Sudanese (2.8%), Yemeni (2.8%), Indian (0.8%), and other (0.8%). Lastly, the region of residence category within Saudi Arabia demonstrates that the highest proportion of participants live in the Western Region, constituting 40.7% of the sample, followed by the Southern Region at 40.3%. Smaller percentages of participants reside in the Northern Region (9.5%), the Central Region (5.9%), and the Eastern Region (3.6%).

**Table 1:** Sociodemographic factors

Sociodemographic factors	n (%)
Age (in years)	
18 - 24	82 (32.4%)
25 - 34	80 (31.6%)
35 - 44	44 (17.4%)
45 - 54	27 (10.7%)
55 - 64	12 (4.7%)
>65	8 (3.2%)
Gender	
Male	195 (77.1%)
Female	58 (22.9%)
Educational status	
Non-educated	9 (3.6%)
Primary school	5 (2.0%)
Middle school	17 (6.7%)
High school	67 (26.5%)
University and above	155 (61.2%)
Marital status	
Currently married	100 (39.5%)
Currently single	142 (56.1%)
Divorced/widowed	11 (4.4%)
Employment status	
Employed	141 (55.7%)
Unemployed	112 (44.3%)
Nationality	
Sudanese	7 (2.8%)
Egyptian	4 (1.6%)
Indian	2 (0.8%)
Saudi	228 (90.1%)
Yemeni	7 (2.7%)
Syrian	3 (1.2%)
Other	2 (0.8%)
Region of residence	
Central Region	15 (5.9%)
Eastern Region	9 (3.6%)
Western Region	103 (40.7%)
Southern Region	102 (40.3%)
Northern Region	24 (9.5%)
Wish to experiment	75 (29.6%)
Status symbol	6 (2.4%)
Why do you continue to use smokeless tobacco?	
Relieves stress (including occupational stress)	120 (47.4%)
I think it enhances job performance	57 (22.5%)
Pleasurable	102 (40.3%)
Addicted to it (not able to quit)	49 (19.4%)
Not aware/worried about its harmful effects	36 (14.2%)
Acts as a medication/mouth cleanser	11 (4.3%)
Are you satisfied to use it?	
Yes	76 (30.0%)
No	177 (70.0%)
Do you plan to quit in the next 12 months?	
Yes	190 (75.1%)
No	63 (24.9)

Table 2 presents the prevalence and consumption patterns of SLT among the study participants. All 253 participants (100%) reported having used SLT products, while none indicated non-use (0%). The majority of participants reported that none of their family members use SLT (58.9%), with 18.6% and 22.5% indicating parents and siblings using it, respectively. Among friends, 29.2% reported none of their closest friends using SLT, 52.2% reported fewer than three friends, and 18.6% reported five friends using SLT. The type of SLT used was then explored. Participants indicated using various types, with toombak (33.2%) and shamma (36.0%) being the most prevalent. Other types had lower prevalence rates, such as afdhal (0.8%), nashooq (7.5%), adani (5.9%), qat (12.6%), and majoon (2.4%). The age at which participants initiated SLT usage showed that 19.0% started before the age of 10, 40.3% between 10 and 20, and 40.7% started after the age of 20. In terms of daily consumption frequency, the majority reported consuming SLT 1-5 times daily (72.7%), while 27.3% reported consuming it 5-10 times or more. The participants' reasons for initiating SLT use were diverse. Influences from parent/relatives (19.0%) and peer pressure/friends (33.6%) were commonly cited, as were reasons like using SLT as an alternative to smoking (32.0%), for mood improvement (30.8%), increased activity (13.0%), adherence to customs and tradition (6.3%), stress reduction (15.8%), appetite reduction (4.7%), and experimentation (29.6%). Continuation of SLT use was attributed to various factors, including stress relief (47.4%), perceived enhancement of job performance (22.5%), pleasure (40.3%), addiction (19.4%), lack of awareness/worries about harmful effects (14.2%), and using it as a medication/mouth cleanser (4.3%). Regarding satisfaction with SLT use, 30.0% of participants expressed satisfaction, while 70.0% were not satisfied. Lastly, participants' intention to quit using SLT within the next 12 months was explored. A substantial proportion (75.1%) indicated a plan to quit, while 24.9% reported no such intention.

**Table 2:** Prevalence and consumption of smokeless tobacco

Questions	n (%)
Have you ever used smokeless tobacco products?	
Yes	253 (100.0%)
No	0 (0.0%)
Does anyone among your family members use smokeless tobacco?	
No	149 (58.9%)
Parent	47 (18.6%)
Sibling	57 (22.5%)
Does anyone among your five closest friends use smokeless tobacco?	
No	74 (29.2%)
Less than 3 friends	132 (52.2%)
All 5 friends	47 (18.6%)
What type of smokeless tobacco do you use?	
Toombak	84 (33.2%)
Shamma	91 (36.0%)
Paan masala (ghutka)	4 (1.6%)
Afdhal	2 (0.8%)
Nashooq	19 (7.5%)
Adani	15 (5.9%)
Qat	32 (12.6%)
Majoon	6 (2.4%)
What was your age when you first started using smokeless tobacco?	
Less than 10 years old	48 (19.0%)
Between 10 and 20 years old	102 (40.3%)
More than 20 years old	103 (40.7%)
How frequently do you consume daily?	
1 to 5 times	184 (72.7%)
5 to 10 times and higher	69 (27.3%)
Why did you begin using smokeless tobacco?	
Parent /relative’s influence	48 (19.0%)
Peer pressure/friend’s influence	85 (33.6%)
Inspired by role model/celebrity	22 (8.7%)
Alternative to smoking	81 (32.0%)
Improves mood	78 (30.8%)
Increases activity	33 (13.0%)
Customs and tradition	16 (6.3%)
Decreases stress	40 (15.8%)
Wish to reduce appetite	12 (4.7%)
Wish to experiment	75 (29.6%)
Status symbol	6 (2.4%)
Why do you continue to use smokeless tobacco?	
Relieves stress (including occupational stress)	120 (47.4%)
I think it enhances job performance	57 (22.5%)
Pleasurable	102 (40.3%)
Addicted to it (not able to quit)	49 (19.4%)
Not aware/worried about its harmful effects	36 (14.2%)
Acts as a medication/mouth cleanser	11 (4.3%)
Are you satisfied to use it?	
Yes	76 (30.0%)
No	177 (70.0%)
Do you plan to quit in the next 12 months?	
Yes	190 (75.1%)
No	63 (24.9)

Table 3 presents participants' awareness of oral cancer and their perceptions related to SLT tobacco usage. Participants' awareness of the harmfulness of SLT compared to smoking tobacco was investigated. The responses indicate that 40.3% of participants believed that SLT is less harmful, while 28.1% disagreed and 31.6% were unsure. In terms of understanding nicotine content, 36.0% of participants thought that the nicotine content of SLT is higher than that of cigarettes, 26.9% disagreed, and 37.2% were uncertain. Regarding the potential connection between SLT use and oral cancer, 57.7% of participants believed that it can cause oral cancer, while 14.6% disagreed, and 27.7% were unsure. Participants were then asked about their recognition of signs associated with oral cancer. The majority believed that difficulty chewing/swallowing (40.3%), a sore that does not heal (39.1%), an abnormal mass/lump in the mouth (43.5%), a white/red patch in the mouth (30.8%), and a slow change in voice quality (34.8%) are signs of oral cancer. Beliefs about the preventability of oral cancer showed that 54.2% of participants thought it is preventable, 11.9% disagreed, and 34.0% were unsure. The misconception about oral cancer being contagious was addressed, with 20.9% of participants thinking it is contagious, 46.6% disagreeing, and 32.4% being unsure. Perceptions about the treatability of oral cancer indicated that 47.4% of participants believed it is treatable, 17.4% disagreed, and 35.2% were unsure. Finally, participants were asked about the potential for dependence and addiction resulting from SLT consumption. A significant majority (63.2%) believed that consumption of SLT can lead to dependence and addiction, 11.1% disagreed, and 25.7% were unsure.

**Table 3:** Participants' awareness of oral cancer and their perceptions related to smokeless tobacco usage

Questions	Yes	No	Don't know
	n (%)	n (%)	n (%)
Do you think smokeless tobacco is less harmful than smoking tobacco?	102 (40.3%)	71 (28.1%)	80 (31.6%)
Do you think that the nicotine content of smokeless tobacco is higher than that of cigarettes?	91 (36.0%)	68 (26.9%)	94 (37.1%)
Do you think smokeless tobacco use can cause oral cancer?	146 (57.7%)	37 (14.6%)	70 (27.7%)
Do you think Difficulty chewing/swallowing is a sign of oral cancer?	102 (40.3%)	41 (16.2%)	110 (43.5%)
Do you think a sore that does not heal is a sign of oral cancer?	99 (39.1%)	44 (17.4%)	110 (43.5%)
Do you think an abnormal mass/lump in the mouth is a sign of oral cancer?	110 (43.5%)	39 (15.4%)	104 (41.1%)
Do you think a white/red patch in the mouth is a sign of oral cancer?	78 (30.8%)	55 (21.8%)	120 (47.4%)
Do you think a slow change in voice quality is a sign of oral cancer?	88 (34.8%)	52 (20.5%)	113 (44.7%)
Do you think oral cancer is preventable?	137 (54.1%)	30 (11.9%)	86 (34.0%)
Do you think oral cancer is contagious?	53 (21.0%)	118 (46.6%)	82 (32.4%)
Do you think oral cancer is treatable?	120 (47.4%)	44 (17.4%)	89 (35.2%)
Do you think consumption of smokeless tobacco leads to dependence and addiction?	157 (62.0%)	30 (11.8%)	66 (26.2%)

Table 4 presents the relationship between awareness of SLT as a cause of oral cancer and various sociodemographic factors among the study participants. The relationship between awareness and age group (p = 0.596), gender (p = 1.000), educational status (p=0.074), marital status (p = 0.200), employment status of participants (p=0.183), nationality (p = 0.384), and region of residence (p = 0.139) were not found to be statistically significant based on the p-values. In other words, these sociodemographic factors did not appear to have a significant influence on participants' awareness of SLT causing oral cancer.

**Table 4:** Awareness of smokeless tobacco as a carcinogen with respect to sociodemographic factors

Variable	Do you think smokeless tobacco use can cause oral cancer?			
	Yes	No	Don't know	P- Value
	n (%)	n (%)	n (%)	
Age				
18 - 24	55 (67.1%)	8 (9.7%)	19 (23.2%)	0.596
25 - 34	43 (53.7%)	15 (18.8%)	22 (27.5%)	
35 - 44	26 (59.1%)	6 (13.6%)	12 (27.3%)	
45 - 54	13 (48.2%)	4 (14.8%)	10 (37.0%)	
55 - 64	5 (41.7%)	3(25.0%)	4 (33.3%)	
>65	4 (50.0%)	1 (12.5%)	3 (37.5%)	
Gender				
Male	112 (57.4%)	29 (14.9%)	54 (27.7%)	1.000
Female	34 (58.6%)	8 (13.8%)	16 (27.6%)	
Educational status				
Non-educated	4 (44.4%)	2 (22.3%)	3 (33.3%)	0.074
Primary school	1 (20.0%)	1 (20.0%)	3 (60.0%)	
Middle school	10 (58.8%)	5 (29.4%)	2(11.8%)	
High school	35 (52.2%)	7 (10.4%)	25 (37.3%)	
University and above	96 (61.9%)	22 (14.2%)	37 (23.9%)	
What is your marital status?				
Currently married	56 (56.0%)	15 (15.0%)	29 (29.0%)	0.200
Currently single	87 (61.3%)	20 (14.1%)	35 (24.6%)	
Divorced/widowed	3 (27.3%)	2 (18.2%)	6 (54.5%)	
Employment status				
Employed	75 (53.2%)	25 (17.7%)	41 (29.1%)	0.183
Unemployed	71 (63.4%)	12 (10.7%)	29 (25.9%)	
Nationality				
Sudanese	5 (71.4%)	1 (14.3%)	1 (14.3%)	0.384
Egyptian	2 (50.0%)	0 (0.0%)	2 (50.0%)	
Indian	0 (0.0%)	1 (50.0%)	1 (50.0%)	
Saudi	131 (57.5%)	34 (14.9%)	63 (27.6%)	
Yemeni	5 (71.4%)	1 (14.3%)	1 (14.3%)	
Syrian	3 (100.0%)	0(0.0%)	0 (0.0%)	
Other	0 (0.0%)	0 (0.0%)	2 (100.0%)	
Region of residence				
Central Region	11 (73.3%)	0 (0.0%)	4 (26.7%)	0.139
Eastern Region	5 (55.6%)	2 (22.2%)	2 (22.2%)	
Western Region	54 (52.4%)	13 (12.6%)	36 (35.0%)	
Southern Region	59 (57.8%)	17 (16.7%)	26 (25.5%)	
Northern Region	17 (70.8%)	5 (20.9%)	2 (8.3%)	

## Discussion

Our study aimed to assess people’s knowledge and awareness of SLT effects in Saudi Arabia.

Regarding the sociodemographic characteristics of the participants. The age distribution indicates a relatively young participant population. The male-female ratio is skewed, with males comprising the majority. Moreover, the educational status demonstrates a diverse educational background, with a significant portion having attained university-level education or above. The marital status reveals that a substantial portion of participants are single, followed by married individuals. In terms of employment status, more than half of the participants are employed, while the rest are unemployed. The nationality distribution is predominantly Saudi, reflecting the study’s focus on the local population. The regional distribution within Saudi Arabia indicates a concentration of participants in the Western and Southern Regions. This geographic concentration may influence certain aspects of the study’s outcomes.

Regarding the clinical and lifestyle characteristics, all 253 participants in the study reported using SLT, with none indicating non-use. It’s noteworthy that the majority of participants reported that none of their family members use SLT (58.9%), and the most prevalent types of SLT used were toombak (33.2%) and shamma (36.0%). These findings align with the work of Bakdash et al. (2017), who similarly observed a high prevalence of SLT use in their study population, with specific types like toombak and shamma being commonly used among participants [19]. However, our study contrasts with the research by Yang et al. (2022), which reported a lower overall prevalence of SLT use (4.4%) and indicated a more varied distribution of usage across different types of SLT products [20].

Our study revealed diverse reasons for initiating SLT use, including influences from parents/relatives and peer pressure/friends. This is consistent with the findings of Sharma et al. (2020), who identified similar factors driving SLT initiation [21]. Additionally, our study’s observation of stress relief and perceived job performance enhancement as factors contributing to SLT continuation echoes the work of Lund et al. (2022), who found comparable motivations among their participants [22]. It’s noteworthy that a significant proportion of participants in our study expressed an intention to quit using SLT within the next 12 months. This contrasts with the results of Patel et al. (2019), who found a lower intention to quit among their study population [23].

A substantial portion of participants believed that SLT is less harmful than smoking tobacco, which aligns with the observations of Chaffee and Cheng (2018) who found similar misconceptions among their study participants [24] This is concerning given the potential health risks associated with SLT use. In terms of participants’ understanding of nicotine content, our study mirrors the results of Spangler et al. (2009) who reported a lack of consensus among participants regarding nicotine levels in SLT versus cigarettes [25]. These misunderstandings highlight the need for accurate health education on this topic.

Regarding the perception of the connection between SLT use and oral cancer, our study’s finding that 57.7% of participants believed in the link corresponds with the work of Mu et al. (2021) who also observed a similar awareness level among their participants [26]. However, this contrasts with the lower awareness reported by Khan et al. (2020) in their study population [27]. The beliefs about the preventability of oral cancer (54.2%) in our study align with the findings of Rimal et al. (2019) who reported similar perceptions among their participants [28].

The misconception about oral cancer being contagious, as addressed in our study, is consistent with the observations made by Biswas (2014), who noted misconceptions related to oral cancer transmission in their research [29]. In terms of perceptions about the treatability of oral cancer, our study’s results differ from the observations of Anirudh et al. (2023) who found a higher percentage of participants believing in the treatability of oral cancer [30]. Our study’s findings on the potential for dependence and addiction resulting from SLT consumption (63.2%) resonate with the conclusions drawn by Salvi et al. (2019) who similarly highlighted the risks of dependence associated with SLT use [31].

There was no statistically significant relationship between participants’ awareness of SLT as a cause of oral cancer and various sociodemographic factors. Specifically, the analysis did not find statistically significant relationships between awareness and factors such as age group, gender, educational status, marital status, employment status, nationality, and region of residence. These results are consistent with the research of Niaz et al. (2020), who similarly found no significant associations between sociodemographic factors and awareness of SLT’s link to oral cancer [32]. Conversely, the observations in our study contrast with the work of Madani et al. (2010), who identified significant associations between awareness and some sociodemographic variables [33].

While this study provides valuable insights into the prevalence and consumption patterns of SLT among the study participants, several limitations need to be acknowledged. Firstly, the study’s sample size may limit the generalizability of findings to a broader population. The participants were drawn from a specific geographical region, potentially impacting the representation of SLT use in other regions. Additionally, the reliance on self-reporting may introduce recall bias or underreporting of certain sensitive behaviors. The cross-sectional design of the study prevents the establishment of causal relationships, limiting our understanding of the temporal dynamics of SLT use initiation and its associated factors.

## Conclusions

The findings indicate a significant prevalence of SLT use, with toombak and shamma being the most common types consumed. Most participants indicated their intention to stop using SLT within the next 12 months, despite having diverse reasons for starting and continuing its use. Awareness of the potential harm of SLT use in relation to oral cancer varied among participants, with a notable proportion misconceiving its harmfulness compared to smoking tobacco. These findings contribute to the ongoing efforts to formulate effective public health campaigns and interventions aimed at reducing SLT consumption and its associated health risks. Also, It is a well-known fact that SLT will cause many complications such as tooth decay and leukoplakia, but we did not investigate this issue because our main objective is to assess the awareness of oral cancer among users of SLT. So, we suggest that complications of SLT should be investigated more to determine the prevalence of each one and awareness of it.

## Figures and Tables

**Figure 1:**
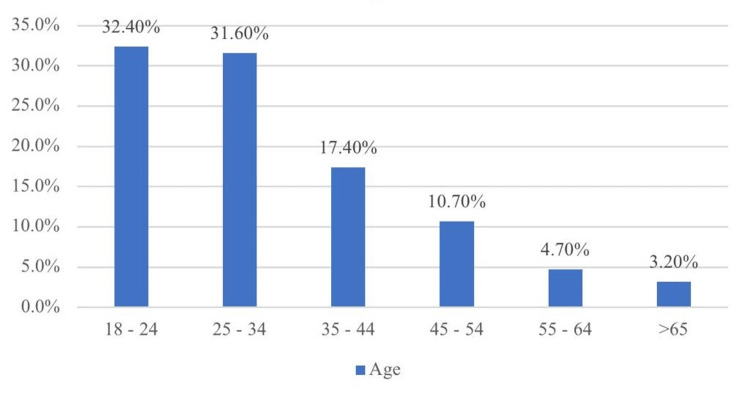
Age

**Figure 2:**
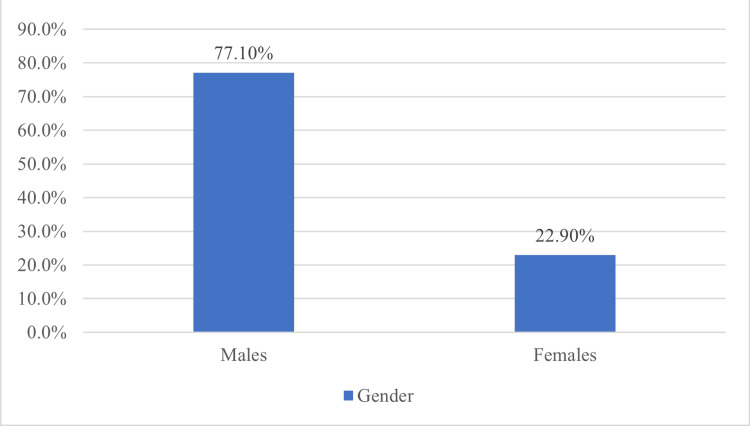
Gender

**Figure 3:**
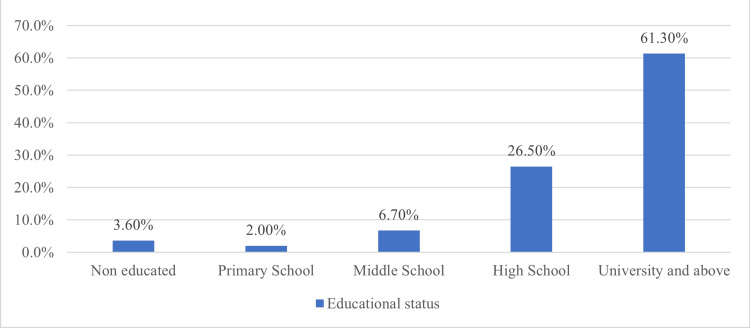
Educational status

**Figure 4:**
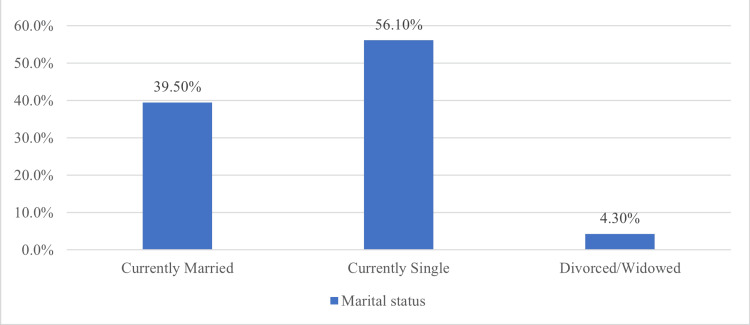
Marital status

**Figure 5:**
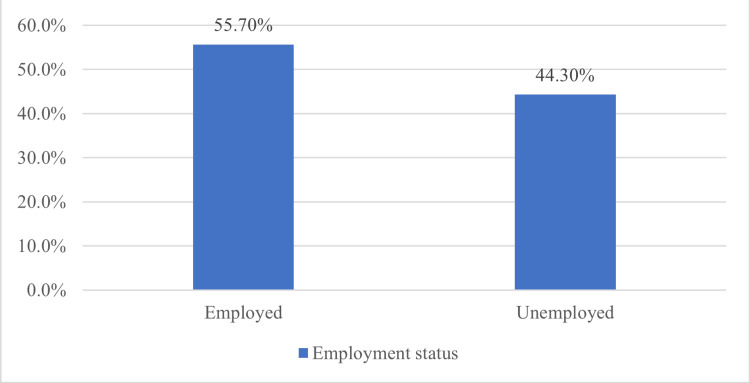
Employment status

**Figure 6:**
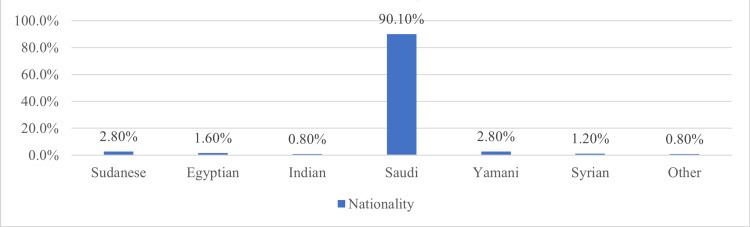
Nationality

**Figure 7:**
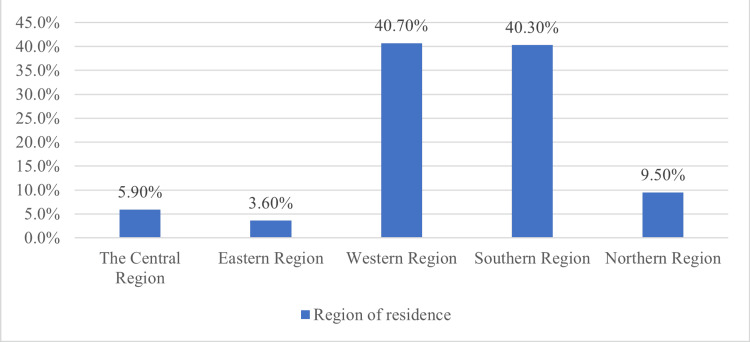
Region of residence

**Figure 8:**
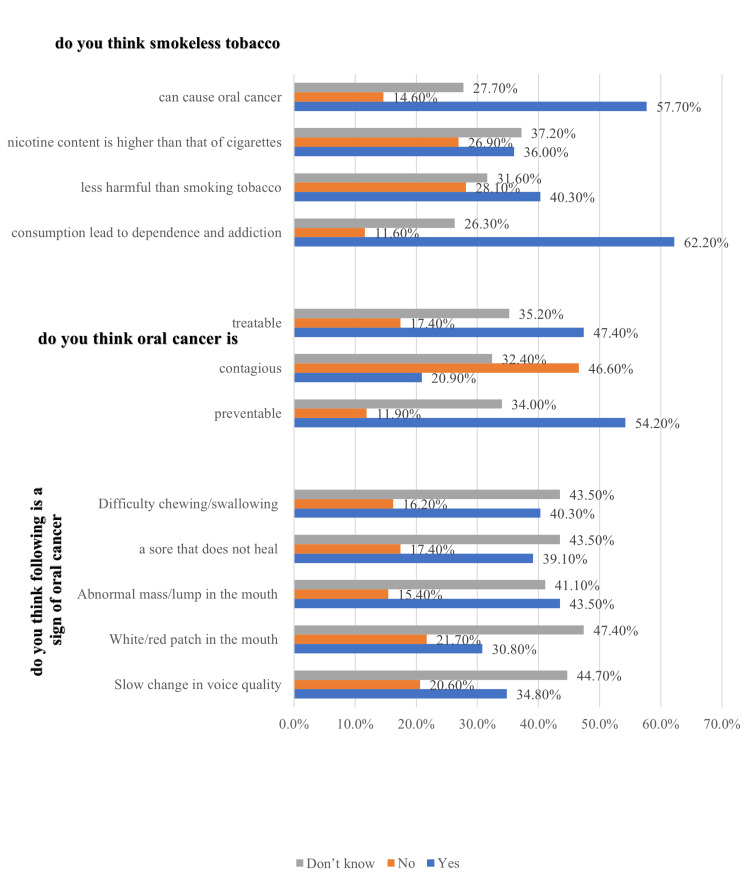
Awareness of oral cancer

## Appendices

Figures 1-8 describe the various findings of our study.
